# Impact of different methods to activate the pediatric mode in automated external defibrillators by laypersons – A randomized controlled simulation study

**DOI:** 10.1016/j.resplu.2022.100223

**Published:** 2022-03-31

**Authors:** Mette V. Hansen, Bo Løfgren, Vinay M. Nadkarni, Kasper G. Lauridsen

**Affiliations:** a Research Center for Emergency Medicine, Aarhus University Hospital, Denmark; b Department of Medicine, Randers Regional Hospital, Denmark; c Emergency Department, Randers Regional Hospital, Denmark; d Department of Anesthesiology and Critical Care Medicine, Children’s Hospital of Philadelphia, United States

**Keywords:** Out-of-hospital cardiac arrest, Pediatric life support, Automated external defibrillators, Simulation

## Abstract

**Introduction:**

Defibrillation with automated external defibrillators (AEDs) for smaller children with out-of-hospital cardiac arrest (OHCA) should be performed using a pediatric mode. This study aims to investigate the easiest and fastest way to activate the pediatric mode on AEDs for pediatric OHCA.

**Methods:**

This randomized, controlled simulation study recruited 90 adult laypersons. Laypersons were randomized to use one of three AEDs with different methods to activate the pediatric mode: a Lifepak CR-T Trainer requiring switch of electrodes, a Phillips Heartstart FR3 Trainer with a “pediatric key”, or a CU Medical IPAD SP1 Trainer with a pediatric button. Laypersons were asked to use an AED on a pediatric manikin and informed that activation of a pediatric mode was recommended.

**Results:**

Activation of the pediatric mode was achieved by 0/30 (0%) participants when switching electrodes (Lifepak CRT), 2/30 (7%) participants when using a key (Phillips FR3) and 18/30 (64%) participants when pushing a button (CU Medical SP1) (p < 0.001). The median (interquartile range) time to first shock among those who activated the pediatric mode were 102 (95–107) in the CU Medical SP1 group and 78 (78–78) in the Phillips FR3 group (p = 0.21). Most participants used the anterior-lateral position for electrodes.

**Conclusion:**

Laypersons’ ability to activate the pediatric mode on AEDs and correctly attach the electrodes was generally poor. More participants were able to activate the pediatric mode by pushing a button when compared to using a key or switching electrodes. Use of the Phillips FR3 AED was associated with faster shock delivery.

## Introduction

Each year thousands of children suffer from out-of-hospital cardiac arrest (OHCA) in the United States and similar numbers are estimated in Europe.1., 2. Early cardiopulmonary resuscitation (CPR) performed by bystanders and the use of an automated external defibrillator (AED) are among the most important factors to improve survival.3., 4. However, user-friendliness of AEDs for defibrillation of children has not been studied.

AEDs are by default set to adult-use delivering high energy shocks. However, when using an AED for children below 8 years of age, defibrillation should be performed with attenuated energy settings of 2–4 J/kg by activating a pediatric mode on the AED.5., 6., 7. This requires laypersons to switch the AED settings from adult to pediatric mode or use pediatric defibrillation electrodes during resuscitation.

Previous studies have reported that user-friendliness is a barrier for swift, effective, and safe defibrillation when using an AED in default adult mode.8., 9. Accordingly, overall bystander AED usage remains low even when the nearest AED is close.^10^ Barriers may be even higher when defibrillating a child because laypersons have to activate the pediatric mode before use.

In different AEDs, the pediatric mode is activated using various methods e.g. by pushing a button, using a special key, or changing the electrodes. These different methods may impact time to electrode placement and time to defibrillation when using an AED by laypersons and healthcare professionals, thus affecting survival outcomes. Currently, the optimal method for activating the pediatric mode remains unknown. This study aims to investigate the easiest and fastest way to activate pediatric mode and defibrillate using an AED when used by laypersons.

## Methods

### Study design

**Fig. 1:**
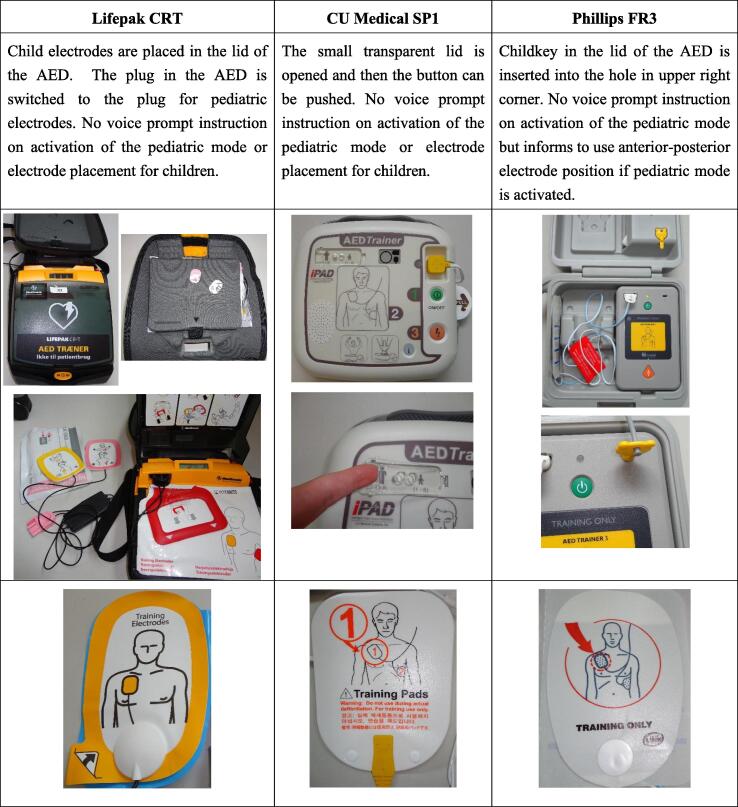
Method to activate pediatric mode and type of electrodes.

This is a randomized, controlled, single blinded simulation study. Participants were randomized (1:1:1) to use one of three different AED models each with a different method to activate the pediatric mode: a Lifepak CR-T Trainer (PhysioControl, Redmond, Washington, USA) where the pediatric mode was activated by change of electrodes, a Laerdal AED Trainer 3/Phillips Heartstart FR3 Trainer (Laerdal Medical, Stavanger, Norway) where the pediatric mode was activated by using a special child-key, and a CU Medical IPAD SP1 AED Trainer (SunTech Medical, Inc., Morrisville, North Carolina, USA) where the pediatric mode was activated by switching a button from “Adult” to “Child 1-8 years”. We chose these 3 AED models after searching for all publicly available AEDs in Denmark as they represent the three currently used methods for activating the pediatric mode among marketed AEDs. Activation of the different pediatric modes are illustrated in Fig. 1. We placed the child electrodes in Lifepak CR-T, so they were visible to the participants. Randomization was performed using random block sizes of 1, 2 or 4 using REDCap that was accessed online using the REDCap webpage by an investigator immediately before the simulation.^11^ Participants were blinded to the exposure and outcomes of the study.

Oral and written consent were obtained from all study participants. In conformity with the Danish National Committee on Biomedical Research Ethics, no ethical review committee approval was required. Participants were informed that they could withdraw their consent at any time.

### Participants

We recruited 90 adult laypersons (≥18-65 ≤ years of age) from schools and teacher’s colleges in the Central Denmark Region. We decided to include employees in schools and students from teacher’s colleges because of their daily contact with children and therefore the possibility of encounter a pediatric cardiac arrest. Exclusion criteria were participants with any kind of healthcare education (e.g. nurse, physician, physio therapist) and/or BLS-instructors.

Participants were asked to complete a questionnaire inquiring on age, sex, education, previous CPR training attendance and time since last CPR training. Participants were also asked to what extent they felt obligated to know about CPR in children due to their work or future work with children. Participants were blinded to the study intervention and endpoints.

### Preliminary study: Overall AED user-friendliness

As this study investigated different methods of activating the pediatric mode in 3 different AED models, we conducted a preliminary study on 30 laypersons to investigate whether the overall user-friendliness and time to first shock were comparable among the three different AED models when using them in default adult mode for laypersons. Methodology and the results of the preliminary study are reported elsewhere.^12^ The preliminary study did not affect the design of this study.

### Cardiac arrest scenarios

Simulations were conducted in available, private rooms at schools or teacher colleges. The participants were informed that a child had collapsed in front of them with a cardiac arrest and that it is recommended to use an AED in pediatric mode when defibrillating a child. No additional instructions on AED operation were provided. Participants were handed the AED and asked to use the AED on the pediatric manikin (Neonatal Resuscitation Model, KOKEN CO., LTD, Bunkyo-ku, Tokyo, Japan), as they would in a real-life situation. The manikin resembled a child being a little bit larger than a usual 1-year old. We chose this neonatal manikin as it resembles a large infant that could not be mistaken for a child 1-8 years of age where it may be chosen not to use the pediatric mode.

The AEDs were set to detect a shockable rhythm and recommend shock delivery. When initiating chest compressions after the first shock, the test was stopped. If the participants did not start chest compressions, the test was stopped 30 seconds after delivering the first shock. All tests were video recorded, and we analyzed time to activation, time to electrode placement, time to first shock and post-shock pause and time to a possible activation of pediatric mode. Due to the nature of the study, the researcher analyzing the videos was not blinded to exposures or endpoints.

The AED electrode placement was digitally photographed, and we assessed whether an anterior-lateral or anterior-posterior placement was used. Attachment of electrodes was defined as placing the whole electrode on the manikin, without tearing it apart or disconnecting the wire from the electrode.

### Endpoints

The primary endpoint was time to first shock measured from the time receiving the AED. Secondary endpoints included: A) Time to turning on the AED. B) Time to activation of pediatric mode and number of participants activating the pediatric mode. C) Time from turning on AED to electrode attachment. D) Correct placement of electrodes. E) Number of participants able to deliver a shock and number of participants able to deliver a shock in pediatric mode. F) Post-shock pause i.e. time from shock delivery to first chest compression. The post-shock pause was defined as time from defibrillation to first compression. All other time measures are defined from start of the scenario (i.e. when the AED was handed to the participant). Activation of the pediatric mode was defined by activating the function by pushing the button (CU Medical SP1), using the child key (Philips FR3), or changing the electrodes (Lifepak CRT). Electrode placement in the anterior-posterior position was defined as correct placement.

### Statistics

Our preliminary study assessed use of AEDs in default adult mode and no previous studies were conducted on the use of pediatric mode for AEDs. Therefore, we chose to include 90 participants (30 in each group). We chose 90 laypersons for this study as it would give a power of 80% to detect an effect size of 0.335 with a significance level of 0.05 using a one-way ANOVA test which we considered to be a clinically important effect size for the study. Normally distributed data were presented as mean ± standard deviation and non-normally distributed data were presented as median (interquartile range (IQR)). Binary data were presented as n (%). Data were analyzed using the Kruskal Wallis test. Binary data were compared using Chi-squared test. All data were analyzed using Stata version 13.0 (StataCorp, College Station, TX, USA). A P-value < 0.05 was considered statistically significant.

## Results

We included 90 adult laypersons from schools and teacher’s colleges. Data were collected from October 2019 to September 2020. Demographics were balanced between the groups except that there was a higher proportion of women in the Phillips FR3 group and 3 participants in this group had performed CPR in real life (Table 1).

**Table 1:** Demographics. Continuous data are presented as median (Q1;Q3) and categorical data are presented as number (%). CPR = Cardiopulmonary Resuscitation. ^a^Missing data for 1 participants. ^b^Missing data for 2 participants.

	**Lifepak CRT** (n=30)	**CU Medical SP1** (n=30)	**Phillips FR3** (n=30)
Gender, female	10 (33%)	9 (30%)	17 (57%)
Age, years	25 (23–42)	26 (22–43)	30 (22–48)
Children, yes	8 (27%)	14 (47%)	15 (50%)
Education:			
Teacher	11 (37%)	13 (45%)^a^	16 (53%)
Student teacher	15 (50%)	15 (52%)^a^	12 (40%)
Other	4 (13%)	1 (3%)^a^	2 (7%)
Type of school:			
Private school	8 (27%)	8 (27%)	10 (33%)
Public school	8 (27%)	8 (27%)	8 (27%)
Teacher’s college	14 (47%)	14 (47%)	12 (40%)
Previous job requiring CPR skills	4 (13%)	5 (17%)	4 (13%)
Previous CPR training	24 (80%)	26 (87%)	25 (83%)
Number of previous CPR courses	2 (1–3)	2 (1–5)	2 (2–3)^b^
Years since last CPR training	2 (0–5)	2 (1–5)	1 (0–3)
Previously performed CPR in real life	0 (0%)	1 (3%)	3 (10%)
Witnessed a real cardiac arrest but not performed CPR	0 (0%)	3 (10%)	4 (13%)
Feel obligated to know about CPR in children due to their work	30 (100%)	30 (100%)	30 (100%)

Continuous data are presented as median (interquartile range) and categorical data are presented as number (%). CPR = Cardiopulmonary Resuscitation. ^a^ Missing data for 1 participants. ^b^ Missing data for 2 participants.

Results are shown in Table 2. All participants in the Lifepak CRT group and the CU Medical SP1 group were able to deliver first shock. In the Phillips FR3 group there was missing data on 1 participant, who did not remove the plastic from the electrodes, thus being unable to deliver the shock and one participant who chose not to use the AED because the manikin resembled a child.

**Table 2:** Results from the simulations. Continuous data are presented as median (interquartile range) and categorical data are presented as number (%). CPR = Cardiopulmonary Resuscitation. ^a^Missing data for 1 participant. ^b^Missing data for 2 participants. ^c^Missing data for 3 participants. ^d^Missing data for 10 participants. ^e^Missing data for 11 participants. Missing data on time to first compression and post-shock pause were mainly due to participants removing the electrodes and not resuming compressions after shock delivery. Unpacking the electrodes was defined as having the electrodes unpacked, in the hands, and ready to attach.

	**Lifepak CRT** (n = 30)	**CU Medical SP1** (n = 30)	**Phillips FR3** (n = 30)	**P-value**
Time to activation of AED (sec.)	9 (6–17)	10.5 (5–16)	6 (4–9)^a^	p = 0.03
Time to unpacking electrodes (sec.)	32 (25–44)	39 (19–52)	15 (11–21)^a^	p < 0.001
Time to electrodes are placed (sec.)	62 (53–76)	82 (74–92)^a^	40 (33–54)^c^	p < 0.001
Attachment of electrodes	28 (93%)	30 (100%)	27 (93%)^a^	
Chooses anterior-lateral placement	27 (90%)	25 (86%)^a^	24 (86%)^b^	
Electrodes overlapping	23 (85%)	24 (96%)^a^	18 (75%)^b^	
Able to deliver shock	30 (100%)	30 (100%)	28 (97%)^a^	
Overall time to first shock (sec.)	81 (73–97)	101 (88–107)^a^	65 (55–78)^c^	p < 0.001
Start compressions after shock-delivery	30 (100%)	28 (93%)	20 (69%)^a^	
Time to first compression (sec.)	90 (83–107)	116 (104–124)^b^	79 (68–93)^d^	p < 0.001
Post shock pause (sec.)	8 (7–10)	16 (14–18)^b^	11 (9–17)^e^	p < 0.001
Able to activate pediatric mode	0 (0%)	18 (64%)^b^	2 (7%)^a^	p < 0.001
Time to activation of pediatric mode (sec.)	No observations	31 (25–45)	67 (57–77)	p = 0.1
Time to first shock (sec.) when activating pediatric mode	No observations	102 (95–107)^a^	78 (78–78)^a^	p = 0.21

**Fig. 2:**
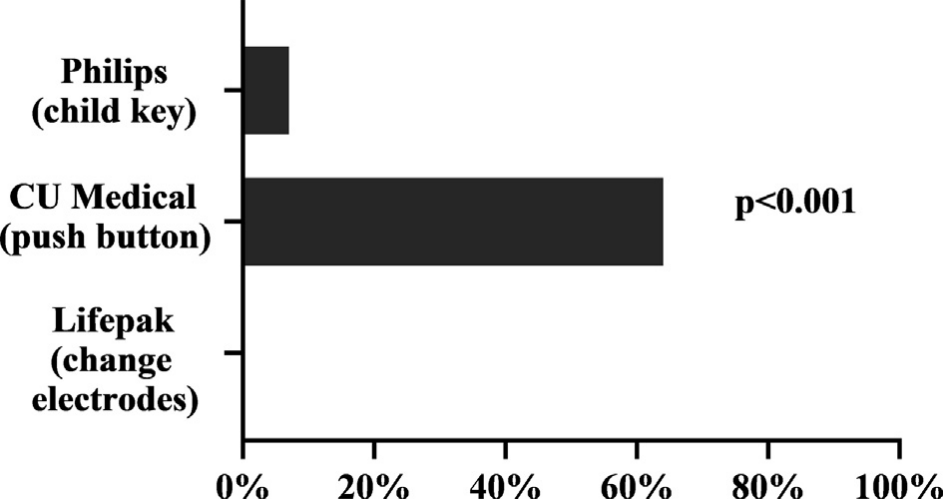
Number of Shows the share of participants in each group able to activate the pediatric mode. There were missing data on 2 participants in the CU Medical group and 1 participant in the Phillips FR3 group.

Activation of the pediatric mode was achieved by 0 (0%) participants when switching electrodes (Lifepak CRT group), 2 (7%) participants when using a key (Phillips FR3 group), and 18 (64%) participants when using a button (CU Medical SP1 group) as shown in Fig. 2 (p < 0.001).

The median (IQR) time to first shock were 81 (73–97) sec. in the Lifepak CRT group, 101 (88–107) sec. in the CU Medical SP1 group and 65 (55–78) sec in the Phillips FR3 group (p < 0.001).

Among participants activating the pediatric mode only, the median (IQR) time to first shock was 102 (95–107) for the 18 participants in the CU Medical SP1 group and 78 (78–78) for the 2 participants in the Phillips FR3 group (p = 0.21). No participants in the Lifepak CRT group activated the pediatric mode.

Overall, 11 (13%) participants used a correct anterior-posterior electrode position, the rest used an anterior-lateral electrode position even though electrodes were overlapping in 65/76 (86%) of cases (Table 2).

## Discussion

This randomized simulation study found that most laypersons were not able to activate pediatric mode or attach electrodes in the anterior-posterior position using the AED instructions alone. Laypersons were better able to activate the pediatric mode by pushing a button as compared to a child-key, and no-one by switching electrodes. Overall, the Phillips FR3 group was fastest at shock-delivery but most participants couldn’t activate the pediatric mode by using the child-key. There was no significant difference in time to shock delivery among participants activating the pediatric mode only.

To our knowledge, this is the first study to investigate activation of the pediatric mode in AEDs. Our findings suggest a general lack of knowledge about the pediatric mode among laypersons. As we assumed that some participants, although working as teachers in schools, may not know about use of pediatric mode, we chose to tell the participants up front that it was recommended to use the AED in pediatric mode. Moreover, as we assumed that switching electrodes to activate the pediatric mode would be difficult, we tried to help participants in the Lifepak CRT group by making the child electrodes visible and showing the part of the electrode stating “child”. Even with this help, no participants were able to activate the pediatric mode. In addition, we recruited teachers/teacher students, as opposed to other laypersons, and made participants aware of the pediatric mode. Furthermore, the vast majority of the laypersons had previous CPR training, some had previous jobs requiring CPR (e.g. work as a lifeguard), and we did not include elderly laypersons that generally have worse CPR skills compared to younger laypersons.^13^ Therefore, we speculate if the number of laypersons activating the pediatric mode may be even lower in a real-life situation with higher levels of stress.

More participants were able to activate the pediatric mode when pushing a button when compared to using a key or changing electrodes. As no participants could activate the pediatric mode by changing electrodes, our findings suggest that change of electrodes in AEDs is not a feasible way of activating the pediatric mode for laypersons, even when making the electrodes visible. Moreover, only 2 participants were able to activate the pediatric mode when using a key. Notably, the key was visible on the front of the AED (Fig. 1) but there was no voice prompt or pictorial instructions to guide the participants, likely making them unable to use this.

Activation of the pediatric mode may have a significant impact on survival outcomes for pediatric OHCA as defibrillation with attenuated energy settings of 2–4 J/kg are known to increase chances of ROSC and minimize myocardial injury from defibrillation for pediatric patients.6., 7., 14. Importantly, a recent study on pediatric OHCA found that bystander use of AEDs was associated with worse survival outcomes.^15^ Although the reason for this finding is unknown, it may be speculated whether inability to activate the pediatric mode by laypersons could explain the inability to show a positive impact on survival outcomes. Thus, future training in use of AEDs on children in cardiac arrest should emphasize how to activate the pediatric mode and the dispatcher should be familiar with the pediatric mode in different AEDs to guide laypersons. Moreover, the voice prompt in future AEDs should be fast and simple with phrases familiar to laypersons, perhaps including a short instruction on how to activate the pediatric mode e.g. “child under 8 years, push the blue button”.

We are unable to make any firm conclusion on how the different methods of activating the pediatric mode affect the time to first shock as so few were able to activate the pediatric mode. As we intended to infer on whether differences in time to first shock was due to overall user-friendliness or the pediatric mode itself, we conducted a preliminary study of other participants using the AEDs in default adult mode.^12^ The preliminary study found that time to first shock was shorter when using the Philips FR3 AED, comparable to our findings in this study. Thus, the findings suggest that the overall difference in time to first shock among the AEDs is due to differences in user-friendliness of general AED-features and not activation of the pediatric mode, supported by the notion that most participants defibrillated without activating the pediatric mode. Features like automatic activation of the AED when opened, easy access to pads and a fast and precise voice prompt seemed to decrease time to first shock.

Time to first shock was comparable (median: 101 vs. 102 sec) for those activating the pediatric mode by pressing a button vs. all participants in the CU Medical group as reported in the preliminary study.^12^ In comparison, the two participants delivering a shock after activating the pediatric mode by using the child key were 13 sec slower than the overall Philips FR3 group.^12^ Although these findings should be interpreted with caution, we find it encouraging that participants delivered shocks with activation of the pediatric mode by pressing a button having comparable time to first shock as those who did not. Ideally, shock delivery should not be delayed by activating the pediatric mode as pre-shock pauses may impact defibrillation success and thereby survival rates and post-cardiac-arrest-outcomes.6., 16., 17., 18..

We found a lack of knowledge about electrode placement on children. Most participants chose to place electrodes in the anterior-lateral position, even though it is recommended to place electrodes in the anterior-posterior position on a small child, so electrodes do not overlap.^6^ Notably, electrode placement on the AEDs are visualized with an anterior-lateral electrode position which may have influenced layperson actions. In most cases (86%) this anterior-lateral placement of the electrodes resulted in the electrodes overlapping each other. Overlapping electrodes was in particular an issue when using the CU Medical SP1 due to larger electrode size when compared to the Phillips FR3 and the Lifepak CRT. Overlapping electrodes might prevent the AED from analyzing heart rhythm and will likely result in less energy delivered through the myocardium, thus decreasing the chance of survival. In fact, it may be questioned whether defibrillation would be successful at all in such cases. Therefore, future training in use of AEDs should emphasize placement of electrodes on children.

## Limitations

This is a simulation study. We used a full-body, realistic neonatal manikin to increase realism. Importantly, this study compared 3 different AED-trainers with 3 different methods of activating the pediatric mode. Our findings from the preliminary study suggest that differences in time to first shock and post-shock pause are due to differences in the overall AED user-friendliness.^12^ We are unable to identify how the different methods of activating the pediatric mode affects the time to activation of pediatric mode and time to first shock with activation of the pediatric mode because so few of participants were able to activate this. The vast majority of participants in our study had previous CPR training and we did not include senior laypersons. Elderlies and untrained laypersons may perform worse.^13^

## Conclusion

Laypersons’ ability to activate the pediatric mode on AEDs and correctly attach the electrodes was generally poor. More participants were able to activate the pediatric mode by pushing a button when compared to using a key or switching electrodes. Use of the Phillips FR3 AED was associated with faster shock delivery.

### CRediT authorship contribution statement

**Mette V. Hansen:** Methodology, Investigation, Formal analysis, Project administration, Writing – original draft, Writing – review & editing. **Bo Løfgren:** Conceptualization, Resources, Methodology, Supervision, Project administration, Writing – review & editing. **Vinay M. Nadkarni:** Conceptualization, Supervision, Writing – review & editing. **Kasper G. Lauridsen:** Conceptualization, Methodology, Investigation, Formal analysis, Project administration, Writing – original draft, Writing – review & editing.

## Declaration of Competing Interest

The authors declare the following financial interests/personal relationships which may be considered as potential competing interests: [The Laerdal AED Trainer 3/Phillips Heartstart FR3 Trainer was provided by Laerdal Medical – Copenhagen, Denmark. The CU Medical IPAD SP1 AED Trainer was provided by Cardiocare Scandinavia. There were no financial, commercial, legal, or professional relationship with any of the organizations.]
